# Host Genetic Factors in Q Fever Susceptibility

**DOI:** 10.3390/pathogens14040394

**Published:** 2025-04-18

**Authors:** José-María Robaina Bordón, José-Luis Pérez-Arellano, Olga Montes-Ares, Alberto Torio-Ruiz, Michele Hernández-Cabrera, Elena Pisos-Álamo, Cristina Carranza-Rodríguez

**Affiliations:** 1 Department of Clinical and Surgical Sciences, University of Las Palmas de Gran Canaria [ULPGC], 35001 Las Palmas de Gran Canaria, Spain; 2 Department of Internal Medicine, Hospital Universitario de Gran Canaria Dr. Negrín, 35010 Las Palmas de Gran Canaria, Spain; 3 University Institute of Biomedical and Health Research [IUIBS], University of Las Palmas de Gran Canaria [ULPGC], 35016 Las Palmas de Gran Canaria, Spain; 4 Hospital Clínico Universitario Virgen de la Arrixaca, 30120 Murcia, Spain; 5 Department of Immunology, Hospital General Universitario de Albacete, 02006 Albacete, Spain; 6 Unit of Infectious and Tropical Diseases, Complejo Hospitalario Universitario Insular, 35016 Las Palmas de Gran Canaria, Spain

**Keywords:** *Coxiella burnetii*, Q fever, host genetic factors

## Abstract

Several indirect findings suggest that host-related factors influence susceptibility to *Coxiella burnetii* infection. We decided to explore the influence of genetic factors related to both innate and adaptive immunity in acute Q fever susceptibility. TLR2 (Arg753Gln) and TLR4 (Asp299Gly, Thr399Ile) polymorphisms, along with HLA-DRB1 alleles, were analyzed for 38 patients with acute Q fever, 38 matched controls, and 121 blood donors. No significant associations were found for TLR polymorphisms. However, HLA-DRB1*04 was more frequent in patients. HLA-DRB1 variants may play a role in Q fever susceptibility, supporting the need for further investigation into their potential implications for vaccination and risk assessment.

## 1. Introduction

Q fever is a globally distributed zoonosis caused by *Coxiella burnetii*, an intracellular gamma-proteobacterium belonging to the Coxiellaceae family [1].

The relevance of this disease lies in several key aspects: (i) it is one of the most common causes of intermediate-duration fever, along with *Rickettsia typhi*, cytomegalovirus, and Epstein–Barr virus [2,3]; (ii) it can lead to severe manifestations, such as endocarditis [1]; and (iii) it represents a major public health problem due to its potential to cause large epidemic outbreaks [4].

The primary route of transmission in humans is the inhalation of contaminated aerosols, which explains its high capacity for dissemination. A discrepancy has been observed between the high seroprevalence of *C. burnetii* in the Canary Islands [5] and the relatively low incidence of clinically diagnosed cases in hospital settings [3,4]. Although microbial factors cannot be ruled out, several indirect findings suggest that host-related factors play a significant role in infection susceptibility and disease severity. In this regard, the persistence of fever in some patients despite appropriate antimicrobial treatment, requiring corticosteroid therapy, suggests the involvement of immunological mechanisms.

To explore this hypothesis, we have decided to investigate the role of TLR2 and TLR4 polymorphisms, as well as the potential influence of HLA alleles, in the development of acute Q fever. Specifically, the aim was to identify whether certain TLR2 or TLR4 polymorphisms, or specific HLA-DRB1 alleles, act as risk or protective factors for the development of acute Q fever.

## 2. Materials and Methods

A total of 38 patients with acute Q fever were prospectively selected. Diagnosis was confirmed using serological methods, based on indirect immunofluorescence antibody titers against phase II *Coxiella burnetii* antigens [IgG > 1:320 and IgM > 1:80] or seroconversion between paired samples collected during the acute and convalescent phases [2 to 4 weeks apart].

An identical number of potentially exposed controls were selected based on the following criteria: (i) similar occupational exposure and (ii) closest familial exposure. Additionally, HLA typing results from 121 blood donors from Gran Canaria were included for comparison.

In both cases and controls, blood samples were analyzed for the following parameters: HLA class II typing [DRB1] and polymorphisms in TLR2 [Arg753Gln] and TLR4 [Asp299Gly, Thr399Ile]. In the control group, the presence of IgG and IgM antibodies against *Coxiella burnetii* was also assessed.

Blood samples were collected, and DNA was extracted using an automated extraction method with the Maxwell 16 system [6]. HLA class II typing was performed using commercial kits [Genprobe] and PCR-SSO techniques on a Luminex platform [7]. Polymorphisms in TLR genes were analyzed using TaqMan probes and real-time PCR [8].

Statistical analysis was performed using IBM® SPSS® Statistics, version 24.0 (IBM Corp., Armonk, NY, USA). Qualitative variables were presented as counts and percentages. Associations between categorical variables were assessed using Pearson’s chi-square test for two proportions, or Fisher’s exact test when ≥20% of the expected values were less than five. A *p*-value < 0.05 was considered statistically significant.

## 3. Results

The study population consisted of 38 patients with a serological diagnosis of Q fever [12 women and 26 men], with a mean age of 39.2 ± 12 years [range: 14–61 years]. Each case was matched with a control [22 women and 16 men] with a mean age of 44.4 ± 12 years [range: 22–79 years].

Regarding the study of innate immunity genes, no significant differences were observed in the three TLR polymorphisms analyzed. All patients and controls carried the GG allele of the TLR2 Arg753Gln polymorphism. For the TLR4 Asp299Gly polymorphism, only one patient carried the CT allele, whereas this allele was detected in four control samples [2.6% vs. 10.5%]. Lastly, the TLR4 Thr399Ile AG allele was identified in five samples from both patients and controls [13.1%].

However, regarding the study of adaptive immunity, significant differences were observed in HLA class II [DRB1 locus] typing between patients and controls [Table 1]. In this cohort, an increased frequency of DRB1*04 was found in patients compared to controls [47.3% vs. 28.9%; *p* = 0.049, cp = NS]. Similarly, the frequency of the DRB1*16 allele was higher in patients than in controls [7.9% vs. 0%; *p* = 0.038, cp = NS]. Conversely, the DRB1*08 allele appeared to have a protective effect, as its frequency was lower in patients compared to controls [2.6% vs. 13.1%; *p* = 0.044, cp = NS].

When comparing the frequencies of these three alleles in patients with those observed in a group of 121 healthy blood donors from the same geographic area, similar trends were noted [Table 2]. The frequencies of DRB1*04 and DRB1*16 were increased in patients, whereas the frequency of DRB1*08 was lower compared to donors. In the case of DRB1*04, the difference between patients and healthy donors reached statistical significance [47.3% vs. 24.8%; *p* = 0.014].

## 4. Discussion

Toll-like receptors [TLRs] are surface proteins found on various immune cells, such as macrophages and dendritic cells, that can recognize structural components of microorganisms. These receptors play a crucial role in pathogen entry into cells and the activation of the immune response. The genes encoding TLRs, particularly TLR-2 and TLR-4, exhibit a high degree of polymorphism. Several studies have linked specific polymorphisms of these genes to variable susceptibility to infectious diseases, including infections caused by classical bacteria, mycobacteria, and other intracellular microorganisms [9]. However, in the present study, no statistically significant association was found between polymorphisms in TLR-2 and TLR-4, which are involved in *Coxiella burnetii* entry into macrophages, and the development of acute Q fever. These findings are consistent with those reported by Everett et al., who also found no statistically significant association between polymorphisms in these genes and susceptibility to Q fever, although their study employed a different methodological design [10]. This result is particularly striking, as multiple studies using animal models have demonstrated a key role of TLR-2 and TLR-4 in the pathogenesis of *Coxiella burnetii* infection [11,12,13].

Antigen presentation to T helper lymphocytes is a key element in the pathogenesis of infectious diseases and plays a crucial role in shaping the host immune response, influencing both protective immunity and susceptibility to infection. Several polymorphisms in the class II HLA system have been associated with increased predisposition to certain infections [14]. In this study, the allele frequency of the HLA-DRB1 locus was analyzed in patients with acute Q fever and compared with a control group. A higher frequency of the DRB1*04 and DRB1*016 alleles was observed in the patient group compared to the control group, whereas DRB1*08 was less frequent among Q fever patients. However, these differences did not reach statistical significance, likely due to the limited sample size. Nevertheless, when comparing allele frequencies with a representative sample of the general population, statistically significant differences were found in the frequency of DRB1*04, suggesting a potential role of this allele in susceptibility to Q fever. There are limited data in the literature regarding the relationship between HLA-DR expression and *Coxiella burnetii* infection. In the study by Helbig et al., HLA-DRB1 frequencies were compared among three groups of Q fever patients: acute, chronic, and chronic fatigue syndrome following Q fever. A higher frequency of HLA-DRB1*11 was observed in the latter group. However, this study did not assess the association between HLA-DRB1 and susceptibility to acute infection [15].

There is growing evidence supporting the relevance of immunogenetic factors in the etiopathogenesis of Q fever. For instance, Wielders et al. associated a specific polymorphism in the IFNG gene (rs1861493) with milder clinical manifestations in cases of acute Q fever [16]. However, most studies have focused on elucidating immunogenetic factors associated with the development of chronic Q fever. In this regard, polymorphisms in IL12B, RAB5A, RAB7A, P2RX7, MAP1LC3A, and ATG5 have been linked to an increased likelihood of progressing to chronic Q fever [17,18].

The identification of patients carrying the HLA-DRB1*04 allele [and potentially HLA-DRB1*08 and HLA-DRB1*16] could facilitate the selection of individuals eligible for potential Q fever vaccination [19]. This approach would be particularly relevant for individuals at high risk of infection or those with an increased predisposition to developing localized forms of the disease, such as immunocompromised people and patients with valvular heart disease or aneurysms. Additional studies with a larger sample size will be required to validate the findings of this study.

## 5. Conclusions

This study found no significant association between TLR2 or TLR4 polymorphisms and susceptibility to acute Q fever. However, differences in HLA-DRB1 allele frequencies—particularly the increased presence of DRB1*04 among patients—suggest a potential role of adaptive immune genetic factors in disease susceptibility. The observed trends warrant further investigation in larger cohorts to clarify the influence of host immunogenetics on acute Q fever risk and to explore their potential relevance for vaccination and risk assessment.

## Figures and Tables

**Table 1:** HLA class II typing study (DRB1 locus) between patients and controls.

DRB1* Alele	Patients 2n = 76 [%]	Controls 2n = 76 [%]	*p*
DRB1*01	7 [9.21]	10 [13.3]	
DRB1*03	10 [13.16]	11 [14.67]	
DRB1*04	18 [26.68]	11 [14.67]	0.049; cp = NS
DRB1*07	12 [15.79]	12 [16]	
DRB1*08	1 [1.32]	1 [6.67]	0.044; cp = NS
DRB1*09	2 [2.63]	0 [0]	
DRB1*10	1 [1.32]	0 [0]	
DRB1*11	4 [5.26]	7 [9.33]	
DRB1*12	1 [1.32]	1 [1.33]	
DRB1*13	12 [15.79]	11 [14.67]	
DRB1*14	0	1 [1.33]	
DRB1*15	5 [6.58]	6 [8]	
DRB1*16	3 [3.95]	0 [0]	0.038; cp = NS

cp: corrected *p* value; NS: not statistically significant.

**Table 2:** HLA class II typing study (DRB1 locus) between patients and blood donors.

DRB1* Alele	Patients 2n = 76 [%]	Blood Donors 2n = 242 [%]	*p*
DRB1*01	7 [9.21]	20 [8.26]	
DRB1*03	10 [13.16]	26 [10.74]	
DRB1*04	18 [26.68]	30 [12.4]	0.014
DRB1*07	12 [15.79]	30 [12.4]	
DRB1*08	1 [1.32]	8 [3.31]	0.177
DRB1*09	2 [2.63]	0 [0]	
DRB1*10	1 [1.32]	4 [1.65]	
DRB1*11	4 [5.26]	28 [11.57]	
DRB1*12	1 [1.32]	2 [0.83]	
DRB1*13	12 [15.79]	60 [24.69]	
DRB1*14	0	6 [2.48]	
DRB1*15	5 [6.58]	20 [8.26]	
DRB1*16	3 [3.95]	8 [3.31]	0.099

## Data Availability

The datasets generated during and/or analyzed during the current study are not publicly available as they are part of ongoing research and further analyses but are available from the corresponding author on reasonable request.
